# Assessing the reliability and validity of attitudes and confidence scales for the care of women and girls affected by female genital mutilation/cutting

**DOI:** 10.1186/s12889-021-11455-8

**Published:** 2021-07-17

**Authors:** Christina X. Marea, Nicole Warren, Nancy Glass, Crista Johnson-Agbakwu, Nancy Perrin

**Affiliations:** 1 School of Nursing and Health Studies, Georgetown University, Washington D.C., USA; 2 School of Nursing, Johns Hopkins University, Baltimore, MD USA; 3 Southwest Interdisciplinary Research Center, Arizona State University, Phoenix, AZ USA

**Keywords:** Female genital mutilation/ cutting, Female circumcision, Health care provider, Attitudes, Confidence, Validation

## Abstract

**Background:**

Approximately 545,000 women and girls in the USA have undergone Female Genital Mutilation/ Cutting (FGM/C) or have mothers from a country where FGM/C is practiced. Women and girls living with FGM/C in the USA may experience stigma and bias due to their FGM/C, immigration, racial, and language status. Health care provider attitudes toward FGM/C and confidence for related clinical care may affect the quality of care, yet there are no validated instruments to measure these constructs.

**Methods:**

We developed the instruments via review of the FGM/C literature, the development of scale items, expert review, and pre-testing. We validated the instruments using a convenience sample of providers in Arizona and Maryland. We used exploratory factor analysis (EFA) to confirm factor structures, and compared scores between known groups to assess validity.

**Results:**

The EFA revealed a two-factor solution for attitudes, including subscales for *Negative Attitudes* and *Empathetic Attitudes* toward FGM/C and those who practice with Cronbach’s alphas of 0.814 and 0.628 respectively. The EFA for confidence revealed a two-factor solution including *Confidence in Clinical FGM/C Care* and *Confidence in Critical Communication Skills for FGM/C Care* with Cronbach’s alphas of 0.857 and 0.694 respectively.

**Conclusions:**

Health care provider attitudes and confidence toward FGM/C care may affect quality of care and health outcomes for women and girls. Our study describes the rigorous psychometric analysis to create reliable and valid instruments to assess health care provider attitudes and confidence for the care of women and girls who have experienced FGM/C.

**Trial registration:**

ClinicalTrials.gov, NCT03249649. Registered on 15 August 2017. Retrospectively registered.

**Supplementary Information:**

The online version contains supplementary material available at 10.1186/s12889-021-11455-8.

## Background

Female Genital Mutilation/ Cutting (FGM/C), a cultural practice with no health benefits, involves the cutting and/or removal of parts of the external female genitalia or other damage to the female genital organs for non-medical reasons [1]. According to UNICEF and the World Health Organization (WHO), FGM/C is practiced in certain communities across approximately 31 countries in Africa, the Middle East and South Asia, with its prevalence varying dramatically between and within countries [1, 2]. Motivations for the practice are diverse and may relate to beliefs about cleanliness, sexual norms, rites of passage, marriageability and/or group membership [3–5]. The WHO has enumerated 33 possible health complications associated with FGM/C divided into five categories including immediate post-FGM/C, obstetric, gynecologic, sexual, and psychological impacts [1]. The WHO has classified FGM/C into four major types depending on the extent of tissue cut or removed and whether the vaginal opening is also narrowed. While all FGM/C types are associated with health complications, not everyone who has undergone FGM/C will experience them. Women with more extensive or severe cutting tend to have worse morbidity [6]. In 2016 and 2018 respectively, the WHO published *Guidelines on the Management of Health Complications from FGM* and *Care of Women and Girls Living with FGM: A Clinical Handbook* which provide comprehensive guidance on the clinical care for those living with FGM/C [1, 7].

Estimates from 2012 indicate that 545,000 women and girls living in the United States of America (USA) may have either undergone FGM/C or were born to mothers from a country where FGM/C is practiced—an increase of 224% since 1990 [8]. As women and girls from families that practice FGM/C are born in or migrate to settings where the practice is non-normative, they may interact with healthcare providers who are unaware of FGM/C, or who do not have the training and confidence to provide evidence-based care. Even if a health care provider is confident in their ability to provide quality care, they may hold attitudes that create barriers in the patient-provider relationship [9]. Health care providers who encounter a patient with FGM/C may experience a strong affective response including anger toward the practice of FGM/C, or bias toward women who experienced it [10]. USA providers and women with FGM/C may further face challenges with cross-cultural communication exacerbated for those with low-English proficiency, particularly if the provider is not skilled conducting visits via interpreter [11–13]. Disagreements about treatment and difficulties in communication can result in a lack of confidence in and distrust of providers by the women [14, 15]. Some women with FGM/C prioritize minimizing interventions; this may be perceived by providers, who may not have actively listened to the patient’s perspective or priorities, as antagonistic or ignorant [16, 17]. Health care providers may experience frustration when women express distrust or refuse recommended care [12]. Women and girls who have experienced FGM/C deserve quality care, yet evidence suggests that they often do not receive it [16, 18–21].

According to the WHO, *quality care* is effective, efficient, accessible, patient-centered, equitable, and safe [22]. Current studies of health care providers caring for women affected by FGM/C suggest there are deficits in care due to a lack of provider knowledge, training and confidence in their ability to provide appropriate care for FGM/C-related complaints. This may result in ineffective or inequitable care [10, 11, 23]. A number of the adverse health consequences related to FGM/C may be compounded by lack of knowledge of the health care provider; for example, lack of familiarity with defibulation may result in the overuse of cesarean birth, and possible neonatal hypoxia due to a prolonged second stage [24]. A recent meta-synthesis of the birth experiences of FGM/C-affected women in the context of migration revealed that a perception that health care providers do not know how to care for them may result in anxiety and fear [16]. Women with FGM/C may distrust health care providers who they feel lack the knowledge and confidence to provide high quality care for FMG/C related complaints [16, 23, 25–29]. This distrust can result in women with FGM/C being less likely to seek or access reproductive health care [30] or to decline recommended care [12]. Health care providers who are knowledgeable about FGM/C frequently learned while caring for women with FGM/C following licensure, rather than during their pre-licensure education [31, 32].

### Existing measures of attitudes and confidence for FGM/C-related care

Health care provider attitudes and confidence impact the quality of care received by women affected by FGM/C, yet there are few existing instruments to measure these constructs. We conceptualized *attitudes* as the expression of an individual’s tendency of favor or disfavor expressed toward a particular entity to inform our instrument development [33]. The tripartite model of attitudes further explains how attitudes toward a topic (i.e. FGM/C) includes behavioral, cognitive and affective components which may impact clinical care and patient experiences [34].

In drafting our attitudes items, we reviewed existing measures created for use in high resource countries where FGM/C is not normative. Existing measures are characterized by the lack of psychometric assessments to determine their reliability or validity. Authors use no [35, 36] or limited validation procedures including expert review [37–39] and pre-testing [32, 37, 38, 40, 41]. Existing measures tend to include items that focus on cognitive aspects of attitudes including ethical issues related to FGM/C (e.g., whether adult women have the right to elect FGM/C, the respondent considers FGM/C a violation of human rights, parents have the right to elect to choose FGM/C for daughters) [32, 40, 42, 43]. The items assessing attitudes towards ethical issues tended to have agree/ disagree Likert scale type responses. Some items included in existing attitudes measures actually capture knowledge rather than cognitive attitudes (e.g., whether FGM/C is a religious or cultural practice), or contextual questions (e.g., whether FGM/C is legal in their jurisdiction, if there are mandatory child protective services reporting requirements, and whether they are aware of parents who intend to have FGM/C for a daughter locally or by traveling abroad [*vacation-*cutting]*)* [43]. Additional attitudes items asked respondents to state how they *would* respond to a patient affected by FGM/C, a behavioral indicator of attitudes [44].

Existing measures of attitudes tend to focus on attitudes toward FGM/C as a practice, rather than attitudes towards the women, girls, and communities that are affected by or support FGM/C or towards the provision of FGM/C-related care. Negative attitudes toward those affected by FGM/C are important to measure because a health care provider’s negative attitude toward a person on the basis of irrelevant characteristics such as race or language proficiency (also known as implicit bias) has been shown to negatively affect clinical care including diagnosis, treatment decisions, health outcomes, non-verbal behaviors, and patient-provider interactions [45, 46]. Most women and girls affected by FGM/C in the USA are of African, Asian or Middle Eastern descent – groups where 50–75% of individuals surveyed report experiencing bias or discrimination in the health care setting [46, 47]. People of color report reduced experiences of bias when they have a health care provider who shares their racial or ethnic identity [46, 48]. Women and girls who have experienced FGM/C embody multiple identities that, within the USA, can render them vulnerable to bias and discrimination including immigration/ refugee status, low-English proficiency, and being a person of color [9, 45, 49].

We began our development of the confidence items by confirming an operational definition and reviewing existing measures. Self-reported confidence in a particular skill is also termed *self-efficacy* and can explain, in part, the actual performance of that skill [50]. Self-efficacy is the perceived capability to perform a behavior and is a robust predictor of whether someone will engage in a target behavior [51]. We identified only two studies since 2007 that assessed health care provider confidence for FGM/C-related care. Neither study included a conceptual definition of confidence or a rationale for item development [36, 39]. Items in one existing study encompassed multiple concepts, thereby limiting interpretability because it is unclear which component of the item respondents considered in their responses [36]. Despite limitations, we noted the following concepts for potential inclusion in our measure including confidence in discussing defibulation, identification and management of FGM/C, documentation, and counseling [36, 39].

The current literature on health care provider attitudes and confidence would be strengthened by measures that utilize clear conceptual definitions of attitudes and confidence, have undergone psychometric testing, and enable researchers to explore the relationships between attitudes, confidence, and other provider characteristics. In this study, we describe the development and psychometric assessment of novel measures of attitudes and confidence of health care providers caring for women and girls affected by FGM/C.

## Methods

### Instrument construction

Authors CM (Nurse-Midwife) and CJA (OB/GYN) discussed the attitudes- and confidence-related domains that emerged from the review of existing measures described above and considered their own experiences providing care for women affected by FGM/C. We sought to develop attitude items that may affect quality of care for women who have experienced FGM/C including attitudes toward the practice of FGM/C, and the women, girls and communities that are affected by the practice. We included items that expressed both negative and empathetic attitudes. We thought of negative attitudes as those that might influence a provider’s affect, behavior, or thoughts about a women in a manner that would lead to a poor patient experience, discrimination or bias related to their FGM/C status. Empathetic attitudes, and their associated feelings, behaviors, and thoughts, might lead the health care provider to prioritize a patient-centered approach to FGM/C-related care. We wrote items about FGM/C as practice to reflect attitudes of condemnation versus empathy regarding the importance of FGM/C to the woman hypothesizing that condemnation may result in a provider’s negative affect experienced by the women as judgement or discrimination. We wrote items related to women who have experienced FGM/C to explore attitudes of the woman as a victim (who may then be treated paternalistically) or a decision-maker (which might result in improved shared-decision making) [52]. We wrote items about those who practice FGM/C to explore attitudes that those who elect FGM/C are knowingly causing harm (which may result in stigmatization of cultural or ethnic groups) versus electing FGM/C in order to care for the long-term prospects of the child (which may result in providers using a more strengths-based approach to education and counseling).

We determined there were broadly two areas of confidence that directly influenced the effectiveness, and equitable patient-centered delivery of care to women affected by FGM/C: confidence in the provision of clinical FGM/C care, and confidence in the ability to communicate effectively with women who have experienced FGM/C. Ideally, we would directly observe provider care; however, given the ethical and practical challenges, we elected to use self-reported confidence as a proxy measure. Items related to clinical care included identifying FGM/C, determining its type, documenting its presence, and discussing potential complications and management. Critical communication skills for care of women affected by FGM/C include the ability to effectively conduct visits via an interpreter, provide counseling on evidence-based treatment options, maintain rapport with a patient who is declining recommended care, and engage in non-judgmental listening.

### Expert review and pre-testing

Next, we requested that experts in the clinical care of women affected by FGM/C review the proposed items (subsequently referred to as FGM/C experts) to assess the content validity [53]. The FGM/C experts included an obstetrician/ gynecologist, a nurse-midwife, and a pelvic floor physical therapist. We circulated a shared document adding comments and edits that were integrated by CM. Comments included grammatical adjustment, clarifying wording to avoid jargon, and simplifying sentence structure. One attitudes item was dropped because it contained multiple clauses and thus responses were not interpretable. No other items were added or dropped. Next, we conducted a pre-test with a small number of individuals to assess if the items were clear and understandable [54]. Author CM reviewed the instrument with a nurse-midwife and medical resident who are not FGM/C experts. CM inquired what they thought each question was intending to ask to ensure that their interpretation was consistent with our intention. No issues arose during this review, and no further revisions were suggested. The survey included 75 items total, including demographic questions. We then beta-tested the survey on the online platform to assess approximately how long it would take respondents to complete, and to ensure there were no programming errors. A convenience sample of 3 medical students, 2 medical residents, and 2 nurse-midwives with no previous FGM/C-related training in current clinical practice in the Phoenix and Baltimore-DC areas completed the beta-test. The full survey took approximately 7–10 min to complete. No further edits were suggested.

### Study setting

We conducted an online cross-sectional survey of health care providers at the time of registration in a workshop titled “Optimizing Care for Women and Girls Affected by FGM/C” in the greater Phoenix and Tucson, Arizona and Baltimore, Maryland areas. The Baltimore area includes significant numbers of immigrants from Sudan, Ethiopia, and Eritrea, while Arizona has resettled a large number of Somali refugees [55, 56]. The FGM/C prevalence (74–98%) in these countries are among the highest in the world, and the FGM/C type is more often type 3 which is the most FGM/C type [57]. This workshop and study were conducted as part of a multi-phase study that aimed to identify and address gaps in care for women and girls affected by Female Genital Mutilation/Cutting (FGM/C). This Office of Women’s Health grant funded programs in various US cities with our primary grant site at Arizona State University focusing on health service needs in greater Phoenix and Tucson. The study team members included researchers from Johns Hopkins University in Baltimore, Maryland thus the inclusion of that region.

### Recruitment and study population

Health care providers were invited to register for the workshop and complete the survey via emails that were distributed to list-servs at 14 health care institutions in Phoenix and Tucson, Arizona metropolitan areas. Arizona list-servs included between 80 and 400 contacts. The survey was broadly distributed within the Johns Hopkins Health System and Johns Hopkins University Schools of Medicine, Nursing and Public Health as well as to professional organizations in the greater Baltimore, Maryland and Washington D.C. area. The primary list-serv contacts included nursing and residency training program directors, medical directors, nursing and medical faculty, and hospital department chairs, and points of contact for local chapters of professional organizations such as AWHONN, ACNM, and ACOG. Electronic consent was obtained from all participants.

The study population included health care providers who registered for the workshop and completed the online survey at the time of registration. This group included physicians, residents, nurses, nurse-practitioners, nurse-midwives, physicians assistants, mental health workers (including psychologists, psychiatrists, and social workers), and students in the health professions.

### Measures

The survey began with items assessing demographic, clinical practice, and previous FGM/C related clinical experience. The survey then included a 33-item checklist assessing awareness of health complications of FGM/C based on complications identified by the 2016 WHO Guidelines, followed by our newly developed attitudes and confidence scales [1]. *Please see* Additional File 1*– FGC KAP Survey for the full survey.* The *Health Care Providers Attitudes Toward FGM/C and Those Who Practice* scale included twelve items total, with five that assessed negative and seven that assessed empathetic attitudes. See Table 1 for all Attitude items. The *Health Care Providence Confidence in FGM/C Care* measure included five items that assessed FGM/C-related clinical care and three items that assessed confidence in communication for FGM/C care, including listening, counseling, and interpreter use. See Table 2 for all confidence items*.* For both the *Attitudes* and *Confidence* scales, participants were asked to read each statement and then mark their level of agreement on a four-point Likert-scale from “4 = Strongly Agree” to “1 = Strongly Disagree”.

**Table 1:** Health Care Providers Attitudes - Items

1	FGM/C is a violation of human rights
2	Communities that practice FGM/C are oppressive towards women.
3	Health Care Providers who perform any form of FGM/C, including symbolic nicking, should be charged with a crime.
4	Parents who have their daughter circumcised are abusing them.
5	Women who have undergone FGM/C are victims of an oppressive cultural practice.
6	Symbolic nicking or cutting of the female genitalia is an effective way to reduce the harm of FGM/C compared to more extensive procedures.
7	Communities that practice FGM/C are honoring an important cultural tradition.
8	Adult women have the right to undergo FGM/C.
9	Parents who have their daughter circumcised are protecting her future marriage prospects.
10	Women who have undergone FGM/C are empowered agents.
11	Parents have the right to have their daughters circumcised (undergo FGM/C).
12	Health care providers should perform reinfibulation (re-closing of the vulvar scar following childbirth) if the woman requests it.

**Table 2:** Health Care Provider Confidence - Items

1	On inspection of the female genitalia, I can identify a woman with FGM/C
2	On identification of a woman with FGM/C, I can assign the appropriate WHO Type classification
3	On identification of a woman with FGM/C, I can appropriately code a visit to document the presence and type of FGM/C using ICD-10 and CPT codes
4	Conduct an effective reproductive/sexual health history via an interpreter
5	Respond to the health concerns of women with FGM/C by engaging in non-judgmental listening
6	Counsel women on the possible complications she may experience related to FGM/C
7	Discuss defibulation with pregnant women who have undergone Type 3 FGM/C in a culturally sensitive manner
8	Create a positive therapeutic relationship with a patient who refuses a recommended procedure

### Statistical methods

We used SPSS version 26 for the analysis. We examined construct validity for each scale using exploratory factor analysis (EFA) with principal axis factoring and oblimin rotation. Factor loadings above 0.40 were considered as loading on a given factor, and those below were considered for revision or elimination from the scale. We reviewed the items loading on each factor to ensure they comprised interpretable constructs and distinct subscales. We used Cronbach’s alpha to assess the reliability of each subscale.

We assessed validity by comparing known groups using independent t-tests; *p*-values < 0.05 were considered significant. For the attitude scales, we tested the a priori hypotheses that health care providers 1) with previous FGM/C training; 2) who have ever cared for a patient affected by FGM/C; 3) who identify as nurses, social workers, or mental health specialists; 4) whose clinical practice focuses on women’s health; 4) who are of color; and 6) who are women will have scores that reflect less negative attitudes and more empathetic attitudes toward FGM/C and those who have experienced FGM/C. We were unable to identify existing studies that performed regression analysis on factors associated with different health care provider attitudes toward FGM/C in the diaspora setting. Thus, our hypotheses are based on the underlying assumption that increased exposure to FGM/C in the form of training or care of women may increase empathetic attitudes, mitigating the negative attitudes towards FGM/C that may result from strongly negative social norms in the diaspora setting. We hypothesized that nursing, social work, and mental health providers would score higher on empathy, and lower on negative attitudes because their professional competencies and training place an emphasis on empathy as a skill [58–60]. We further hypothesized that people of color and women would have more empathetic and less negative attitudes based on literature that has suggested that clients prefer racial and gender concordance, and that it may result in improved communication [61].

For the confidence scale, we tested the a priori hypotheses that those 1) who have had previous FGM/C training; 2) who have cared for a patient affected by FGM/C; and 3) whose clinical practice focuses on women’s health will have scores that reflect greater confidence in the care of those affected by FGM/C.

## Results

### Enrollment and participant characteristics

A total of 796 individuals registered for training events in Arizona and 101 in Maryland for 897 possible survey participants. A total of 368 participants initiated the online survey, of whom 14 did not provide any responses following the informed consent screen. The remaining 354 participants completed the online survey for a response rate of 39.5%. Our survey invited participants to report their gender (male, female, transgender, other) and race/ ethnicity (including a free-text option to self-define); participants could elect not to respond to these items. Exploratory factor analysis requires that all participants have completed all items to be included in the analysis. For the Attitudes scale, 291 (82.2%) participants completed all items and were included in the EFA. For the Confidence scale, we limited analysis to health care providers who are licensed independent providers (LIP), defined as physician or advanced practice nurses (*n* = 169, 47.7% of the total sample). These providers conduct the outpatient medical care that *Confidence* scale items address. Of the LIPs *n* = 143 (84.6%) completed all Confidence items. There were no significant differences in any participant characteristics between completers and non-completers. Participants for the attitudes EFA sample predominantly reported their gender as female (81.4%), white (67.5%), and had a clinical specialty outside of women’s health (65.0%). Demographics were similar for the attitudes and confidence sub-groups; however, in the subsample for the confidence scale EFA more participants were white (77.6%) and had a women’s health focus in their clinical practice (52.4%). See Table 3 for detailed participant characteristics.

**Table 3:** Participant Characteristics

	All Health Care Providers Attitudes EFA^b^ (*n* = 291)	Licensed Independent Providers Only Confidence EFA^b^ (*N* = 143)
	N (%)	N (%)
Gender		
Female	240 (82.5)	108 (75.5)
Male	37 (12.7)	28 (19.6)
Missing/ Declined/ Transgender/ Other	14 (4.8)	7 (4.9)
Race/ Ethnicity		
Person of Color	97 (33.3)	33 (23.1)
Black/ African American	30 (10.3)	8 (5.6)
Asian	31 (10.7)	15 (10.5)
Latino/ Hispanic; Native American; Other (non-white)^a^	35 (12.0)	10 (6.8)
White	194 (66.7)	109 (76.2)
Clinical Practice		
Licensed Independent Provider	*138 (47.4)*	
Resident	70 (24.1)	72 (50.3)
Physician	38 (13.1)	37 (25.9)
CNM	22 (7.9)	25 (17.5)
NP	8 (3.4)	9 (6.3)
Other Health Care Provider	*153 (52.6)*	–
RN	38 (13.1)	–
Social Work	9 (3.1)	–
Mental Health	8 (2.7)	–
Student	72 (24.7)	–
Other/ Missing	11 (6.2)	–
Women’s Health Specialty		
Yes	86 (29.6)	74 (51.7)
No	189 (64.9)	62 (43.4)
Missing	16 (5.5)	7 (4.9)
Scope of Practice includes BIRTH (Ob/Gyn, Midwife)		
Yes	67 (23.0)	73 (51.0)
No	208 (71.5)	63 (44.1)
Missing	16 (5.5)	7 (4.9)

^a^Due to small sample size for these demographic groups, they were combined to protect participant privacy

^b^Demographics for participants who completed all items for the respective scale

Fewer than half of the sample of health care providers had ever cared for a woman or girl who had experienced FGM/C (44.0%), though among the subsample of licensed independent providers two-thirds had (69.4%). Most participants had not received any formal training regarding FGM/C. See Table 4 for additional details about the FGM/C related clinical experiences of the participants.

**Table 4:** FGM/C Clinical Experiences

	Attitudes EFA All Health Care Providers (*n* = 291)	Confidence EFA Licensed Independent Providers Only (*N* = 143)
Ever Cared for Patient with FGM/C		
Yes	128 (44.0)	99 (69.2)
No	163 (56.0)	44 (30.8)
Previous FGM/C Training		
Yes	101 (34.7)	64 (44.8)
No	190 (65.3)	79 (55.2)

### Results – psychometric testing for attitudes

**Fig. 1:**
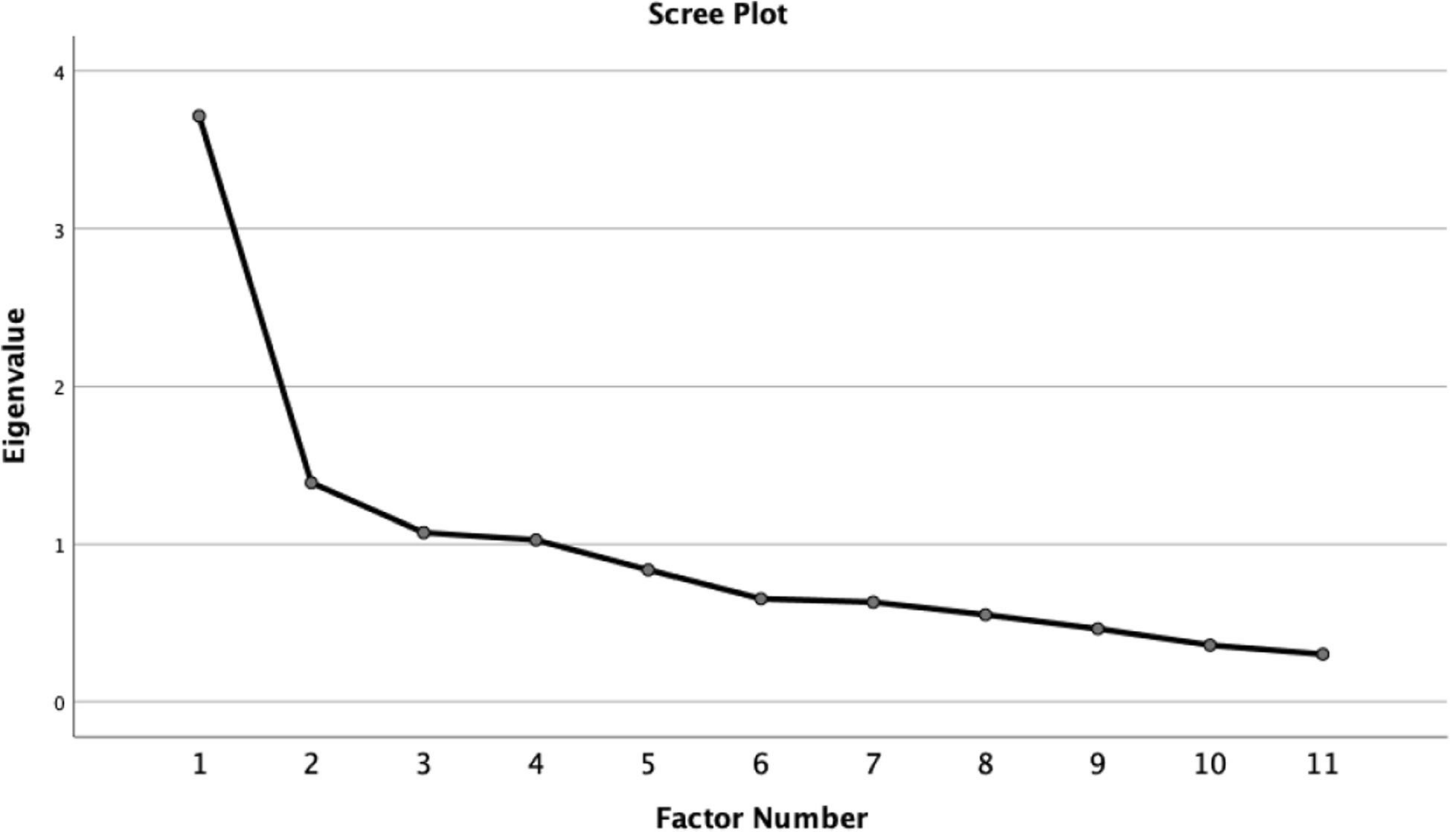
Scree Plot - Attitudes

We began the exploratory factor analysis by reviewing the cases for completeness which demonstrated 3–9.9% missing responses per item. No items were eliminated due to missingness. We reviewed the variance of each item and eliminated one item because it had minimal variance with greater than 93% responding either disagree/ strongly disagree (*Parents have the right to have their daughters circumcised/ undergo FGM/C)*). The lack of variance in this item may be because FGM/C is commonly considered a violation of human rights in the USA. The eigenvalues (first 5 were 3.72, 1.39, 1.07, 1.03, 0.84) suggested that the 11 items formed two factors, which we confirmed using a scree plot visualization to identify the two factors above the break in the plot. See Fig. 1 for the Scree Plot.

All factor loadings were above 0.400 on only one of the two-factors except for *Women who have undergone FGM/C are empowered agents* which had a factor loading of 0.390 and was retained for the final subscale. The two-factor solution accounted for 37.5% of the total variance. Each item loaded cleanly on one of the two factors, except for *Health care providers should perform reinfibulation (re-closing of the vulvar scar following childbirth) if the woman requests it* which did not load on either factor and thus was dropped. This item likely did not perform well in the EFA because many participants may be unfamiliar with the term *reinfibulation*. See Table 5 for the rotated factor loadings for the Attitudes items.

**Table 5:** Rotated Factor Loadings – Attitudes Items

	Negative Attitudes Toward FGM/C and Those Who Practice FGM/C	Empathetic Attitudes Toward FGM/C and Those Who Practice FGM/C
FGM/C is a violation of human rights	**.593**	−.066
Health Care Providers who perform any form of FGM/C, including symbolic nicking, should be charged with a crime	**.455**	−.099
Communities that practice FGM/C are oppressive towards women	**.809**	.101
Parents who have their daughter circumcised are abusing them	**.804**	.039
Women who have undergone FGM/C are victims of an oppressive cultural practice	**.766**	−.041
Symbolic nicking or cutting of the female genitalia is an effective way to reduce the harm of FGM/C compared to more extensive procedures	.024	**.444**
Adult women have the right to undergo FGM/C	−.025	**.440**
Communities that practice FGM/C are honoring an important cultural tradition	−.028	**.557**
Parents who have their daughter circumcised are protecting her future marriage prospects	.043	**.637**
Women who have undergone FGM/C are empowered agents	−.044	**.390**
**Cronbach’s Alpha**	*0.814*	*0.628*
**DROPPED ITEM**		
Health care providers should perform reinfibulation (re-closing of the vulvar scar following childbirth) if the woman requests it	−.121	.244

Items loaded according to whether they addressed negative or empathetic attitudes toward FGM/C and those who are affected by FGM/C (further referred to as *Negative Attitudes* and *Empathetic Attitudes* for brevity). Each subscale has five items. The correlation between the factors is − 0.558 which indicates that the factors have a strong correlation but represent distinct underlying variables that are inversely correlated. The communalities for all items range from 0.173–0.624.

### Principal Axis factoring. Rotation method: Oblimin rotation

#### Reliability

We assessed the reliability of the subscales using Cronbach’s alpha – a measure of internal consistency of the items as they perform in a specific sample. The *Empathetic Attitudes* subscale has a Cronbach’s alpha of 0.628. This meets the 0.60 threshold considered the minimum acceptable for early stages of research [62, 63]. Further, Cronbach’s alpha tends to underestimate the internal consistency of scales with fewer than 10 items such as ours [64, 65]. The *Negative Attitudes* subscale has a Cronbach’s alpha of 0.814 which is considered an acceptable level of internal consistency.

#### Descriptive statistics

We calculated total scores for each subscale by summing scores for each of the five items. Higher scores indicate more negative attitudes and more empathetic attitudes on each scale respectively. See Table 6 for means, standard deviations, minimum and maximum observed scores.

**Table 6:** Attitudes - Descriptive Statistics

	Mean	Std. Dev.	Min	Max	Possible Range
**Negative Attitudes Toward FGM/C**	15.85	2.65	10	20	5–20
**Empathetic Attitudes Towards FGM/C**	11.75	2.37	5	20	5–20

(*n* = 291)

The mean score on the *Negative Attitudes* was 15.85 (SD 2.65) out of 20 indicating that the sample tended to agree or agree strongly with statements that convey negative attitudes toward FGM/C and women affected by FGM/C. The range of scores for the *Negative Attitudes* subscale was between 10 and 20 with a roughly normal distribution of scores. The mean score on the *Empathetic Attitudes* was closer to the mid-range of possible scores (11.75, SD 2.37) indicating that participants tended to agree or disagree with the statements, rather than holding strong opinions on either end of the response range. The range of scores for participants on the *Empathetic Attitudes* subscale included the full possible range from 5 to 20.

#### Known groups validity

We assessed validity by comparing scores between known groups using independent t-tests. There were no significant differences in scores on the *Negative Attitudes* or *Empathetic Attitudes* scales between those who had received training or ever care for patient with FGM/C compared to those without those experiences. Licensed independent providers (MD/ CNM/ NP) had significantly higher scores on the *Negative Attitudes* scale and significantly lower scores on the *Empathetic Attitudes* scale compared with nurses and mental health providers which supports our hypothesis that these providers may have higher empathy scores due to the focus on empathy as a professional skill in their respective professions. Scores were not significantly different on either scale for participants with a clinical focus in women’s health. Male participants and white participants both had significantly higher scores on the *Negative attitudes* scale which supports our hypotheses; however, there were no significant differences in scores on the *Empathetic Attitudes* scale for either of these two groups. See Table 7 for detailed known group validity analysis.

**Table 7:** Criterion Validity – Attitudes

	*N*	Negative Attitudes		Empathetic Attitudes	
		Mean (SD)	*p*	Mean (SD)	*p*
**PREVIOUS FGM/C EXPERIENCES**					
Ever Cared for FGM/C-Affected Patient					
Yes	128	16.05 (2.53)	0.259	11.73 (2.42)	0.943
No	163	15.69 (2.74)		11.75 (2.34)	
Received FGM/C Training^a^					
Yes	101	15.75 (2.41)	0.652	11.96 (2.31)	0.262
No	190	15.90 (2.77)		11.63 (2.40)	
**SCOPE OF PRACTICE**					
Nursing and Mental Health Providers					
Yes	149	15.51 (2.77)	0.031*	12.19 (2.30)	0.001*
No	141	16.18 (2.47)		11.30 (2.36)	
Women’s Health Specialty^a^					
Yes	86	16.00 (2.35)	0.512	11.74 (2.26)	0.915
No	189	15.79 (2.75)		11.78 (2.50)	
**DEMOGRAPHIC**					
Gender					
Male	37	16.62 (3.06)	0.050*	11.22 (2.03)	0.107
Female	240	15.71 (2.55)		11.88 (2.37)	
Race					
White	194	16.12 (2.65)	0.014*	11.57 (2.48)	0.078
Person of Color	97	15.31 (2.59)		12.09 (2.12)	

(*n* = 291)

^a^Unequal variance

*Statistically Significant < 0.05

### Results – psychometric testing for confidence scale

The psychometric analysis of the Confidence scale includes licensed independent providers (physicians, nurse-midwives, and nurse-practitioners). We began the factor analysis by reviewing the cases for completeness which demonstrated 4–9% missing responses per item. We reviewed the item variance and found that *Respond to the health concerns of women with FGM/C by engaging in non-judgmental listening* and *Create a positive therapeutic relationship with a patient who refuses a recommended procedure* had minimal variance with > 91% selecting agree or strongly agree. We retained these items for the EFA because they addressed important aspects of quality care related to communication.

**Fig. 2:**
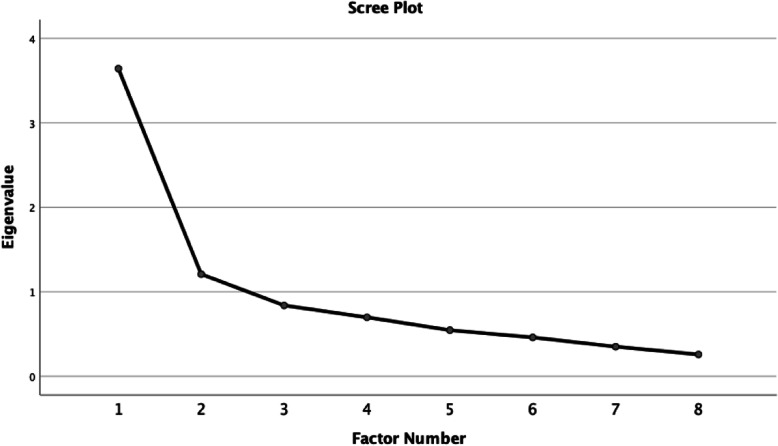
Scree Plot - Confidence

The EFA resulted in a 2-factor solution, which accounted for 50.87% of all variance. The eigenvalues (first five were 3.64, 1.21, 0.84, 0.70, 0.55) suggested that the eight items form two factors, which we confirmed using the scree plot to visualize two factors above the break in the plot. See Fig. 2.

All items had factor loadings over 0.400 and each item loaded on a single factor. See Table 8 for rotated factor loadings for the confidence items. The communalities range from 0.223–0.823.

**Table 8:** Rotated Factor Loadings – Confidence Items

	Confidence in Clinical FGM/C Care	Confidence in Critical Communication Skills for FGM/C Care
On inspection of the female genitalia, I can identify a woman with FGM/C	**.569**	0.166
On identification of a woman with FGM/C, I can assign the appropriate WHO Type classification	**.970**	−0.185
On identification of a woman with FGM/C, I can appropriately code a visit to document the presence and type of FGM/C using ICD-10 and CPT codes	**.736**	−0.064
Counsel women on the possible complications she may experience related to FGM/C	**.512**	0.380
Discuss defibulation with pregnant women who have undergone Type 3 FGM/C in a culturally sensitive manner	**.695**	0.183
Conduct an effective reproductive/sexual health history via an interpreter	0.285	**.463**
Respond to the health concerns of women with FGM/C by engaging in non-judgmental listening	0.080	**.747**
Create a positive therapeutic relationship with a patient who is refuses a recommended procedure	−0.094	**.670**
***Cronbach’s Alpha***	*0.857*	*0.694*

Principal Axis Factoring. Rotation Method: Oblimin Rotation

The first subscale addresses items related the provision of clinical care of women who have experienced FGM/C, titled *Confidence in Clinical FGM/C Care*. The second subscale includes items that assess the health care provider’s confidence in communication skills titled *Confidence in Critical Communication Skills for FGM/C Care*.

### Reliability

The Cronbach’s alpha coefficient for the two confidence subscales were 0.857 for the *Confidence in Clinical FGM/C Care* and 0.694 for *Confidence in Critical Communication Skills for FGM/C Care,* respectively, again indicating acceptable levels of reliability.

### Descriptive statistics

We calculated scores for the subscales by summing the scores for each item. The subscale *Confidence in Clinical FGM/C Care* has five items on a four-point Likert scale from strongly disagree = 1 to strongly agree = 4 for a possible score range of 5–20. The subscale addressing *Confidence in Critical Communication Skills for FGM/C Care* has three items on the same four-point Likert scale for a possible range of 3–12. Higher scores indicate more confidence. The correlation between the two factors is 0.461 which indicates that the subscales are positively correlated but do measure distinct latent constructs. See Table 9 for descriptive statistics.

**Table 9:** Descriptive Statistics – Confidence Scales

	Mean	Std. Dev.	Range (possible)	Min	Max
**Confidence in Clinical FGM/C Care**	11.52	2.90	5–20	5	20
**Confidence in Critical Communication Skills for FGM/C Care**	9.08	1.35	3–12	3	10

(*n* = 143)

The mean score on the *Confidence in Clinical FGM/C Care* subscale (11.52) indicates that participants generally endorsed a moderate level of confidence. The distribution of scores on this subscale is approximately normally distributed with slightly more participants reporting lower levels of confidence. The mean score on the *Confidence in Critical Communication Skills for FGM/C Care* subscale (9.08) indicates somewhat higher confidence in communication skills compared to clinical skills for FGM/C. The distribution of scores for this subscale is approximately normally distributed.

### Known group validity – confidence scales

We assessed validity by comparing scores between known groups using independent t-tests. Health care providers who have ever cared for someone affected by FGM/C, have ever received FGM/C training, whose scope of practice includes a women’s health specialty, people of color, and women rated their *Confidence in Clinical FGM/C Care* significantly higher than the comparison groups. Health care providers who have ever cared someone who has experienced FGM/C and those with a scope of practice focused on women’s health had significantly high scores on the *Confidence in Critical Communication Skills for FGM/C Care* scales than the comparison groups. There was no significant difference in *Confidence in Critical Communication* scores for those who had received previous training related to FGM/C. See Table 10 for further details on validity testing.

**Table 10:** Criterion Validity – Confidence Subscales

		Confidence in Clinical FGM/C Care		Confidence in Critical Communication Skills forFGM/C Care	
	N	Mean (SD)	*p*	Mean (SD)	*p*
**PREVIOUS FGM/C EXPERIENCES**					
Ever Cared for FGM/C-Affected Patient					
Yes	99	12.51 (2.59)	0.000*	9.32 (1.23)	.001*
No	44	9.27 (2.25)		8.53 (1.47)	
Received FGM/C Training					
Yes	64	12.32 (2.38)	0.002*	9.20 (1.29)	.347
No	79	10.88 (3.12)		8.99 (1.40)	
**SCOPE OF PRACTICE**					
Women’s Health Specialty					
Yes	74	12.42 (2.58)	> 0.000*	9.35 (1.27)	.010*
No	62	10.34 (2.81)		8.76 (1.39)	

(*n* = 143)

*Statistically Significant < 0.05

## Discussion

In this study, we have constructed measures of health care provider attitudes and confidence for the care of women and girls affected by FGM/C and assessed their validity and reliability. We utilized a conceptual approach to our item development by first defining attitudes and confidence, and then framing which types of attitudes (toward the practice and those affected on a spectrum of negative to empathetic), and confidence (clinical skills and communications skills) were important when considering their effect on quality of care based on review of qualitative studies and existing measures. Our attitudes scales include some items that are similar to existing measures (harm reduction, whether adult women have the right to undergo FGM/C electively, human rights) and adds novel items to assess attitudes towards communities that practice FGM/C and affected women. Our attitudes scales include controversial topics such as reinfibulation because of the ongoing ethical and legal debate about the right of adult women to elect surgical alterations to their genitalia, including what and who determine what constitutes FGM/C versus Female Cosmetic Genital Surgery [9]. Overtime, items assessing attitudes toward controversial topics may provide researchers with an understanding of how attitudes are shifting, particularly in response to interventions or broader social change.

The psychometric properties of the confidence subscales were strong overall. The subscales have a clear factor structure, and acceptable reliability. The validity of the *Confidence in Clinical FGM/C Care* is supported by the significance of the hypothesized group differences. The validity for the *Confidence in Critical Communication Skills for FGM/C Care* is supported by the significance of two of three hypothesized group differences. While we did not find a significant difference in confidence scores for those with and without previous FGM/C training in the *Confidence in Communication* subscale, this could potentially be a result of training programs focusing more on clinical skills than communication skills. There is very limited published data on existing trainings for FGM/C, and none we reviewed evaluated training effectiveness to improve communication skills.

The attitudes subscales also have clear factor structures, and the reliability of the *Negative Attitudes* subscale is acceptable. The reliability of the *Empathetic Attitudes* subscale falls just below the acceptable threshold. Cronbach’s alpha has some limitations as a measure of reliability. It measures the internal consistency of the items within a specific sample, where more heterogenous populations tending to increase the Cronbach’s alpha. Given that our sample was self-selected, they may be more homogenous in terms of attitudes toward FGM/C compared to the general population of health care providers. Our subscales also have a relatively small number of items (< 10), which can lower the Cronbach’s alpha. The reliability of the scales should be further assessed in a random sample of health care providers. Future research should assess the reliability of these scales with a more diverse study population that varies in relation to demographic variables such as race, gender, and political affiliation; travel experience; and whether FGM/C has been practiced within their family or close contacts. Further validation of the scale could also include assessment of concurrent validity. Though there are no validated measures of attitudes related to FGM/C, comparing participants’ scores on our scale with scores on validated measures of empathy and implicit bias may further inform the validity of the attitude scales.

There are some important differences between our subscales and existing measures. We did not include attitudes items that assessed whether participants believe FGM/C is a religious practice or whether they knew their legal or reporting requirements because these questions address knowledge rather than attitudes [42, 43]. Our confidence scales included similar concepts compared to existing measures such as items addressing identification and documentation of FGM/C; however, our scales included more items regarding communication and counseling skills [36, 39]. Our scales are consistent with existing measures for attitudes and confidence due to the inclusion of items about defibulation and reinfibulation [32, 36, 40].

Within the USA, many women and girls affected by FGM/C are migrants or refugees from Africa, Asia, or the Middle East who may have low-English proficiency – all variables that may contribute to experiences of bias, discrimination, and lower quality of care. The historical legacy of slavery and racism in the USA, and in particular the “othering” of African women’s bodies, may implicitly influence the patient-provider interaction, counseling and decision-making [9]. Health care providers must take responsibility for communicating attitudes that promote positive patient/provider interaction so that FGM/C affected women do not experience bias or discrimination during a clinical encounter. Our scales are an important step to assess health care provider attitudes toward FGM/C and those affected by the practice, and eventually to explore how their attitudes are related to patient experiences of care. The new attitudes subscales may allow researchers and educators to explore whether health care providers who experience more empathic and less negative attitudes provide higher quality care to women affected by FGM/C. While empathy does tend to improve patient-provider communication, it is important to assess if health care provider empathy moves toward endorsement of harm reduction approaches to addressing FGM/C versus elimination of the practice [66]. Future research should explore empathetic attitudes toward FGM/C as a practice that adult women may elect versus a practice conducted on a child. Some health care providers maintain that the less extensive forms of FGM/C, such as symbolic nicking, should be permitted in place of more extensive cutting to minimize harm [66–68]. By including items that assess strongly empathetic attitudes toward FGM/C, researchers and educators may be able to provide additional education to health care providers in the diaspora regarding the possible physical harms that may result from the different forms of FGM/C, and the ethical arguments that support the opposition to FGM/C on a girl child because it causes irreversible change, physical harm, and that a child cannot consent to the practice [69]. Some argue that minors (child with male, female, or intersex genitalia) must be protected from bodily harm, which includes nontherapeutic genital surgery or cutting (including male circumcision) [70]. Though these attitudes may be less common in the USA, our scales may detect the range of empathetic attitudes.

In our study sample, previous training in FGM/C was not a statistically significant predictor of increased *Confidence in Communication for FGM/C*. This may be because existing FGM/C trainings do not focus on communication skills, or if they do, may not provide an opportunity to practice those skills in order to measurably improve provider confidence [71]. Future trainings should include informational content about FGM/C (history and context, identification, documentation, complications, and management for women affected by FGM/C) to build provider confidence in their knowledge to provide clinical care for FGM/C. Trainings should include opportunities for simulation, reflection, and engagement to practice applying knowledge, communications skills, and build awareness of how attitudes may affect the quality of care delivered to those affected by FGM/C [72]. Communication skills training must include interpreter use, including its benefits, potential risks, impact on provider-client rapport and trust, and ethical considerations [73]. It is imperative that trainings for health care providers caring for women affected by FGM/C, and in fact any marginalized population, include instruction in reflective practice. Reflective practice involves deepening one’s understanding of self, other, and situation at the cognitive, behavioral, and affective levels [74]. Broad health workforce education and training to improve the care of women affected by FGM/C, including effective evaluation of education and training interventions, is an important public health priority [72].

Some limitations of our study include geographic restriction of the study population to the greater Phoenix, Tucson, and Baltimore areas and a low response rate by the study population. Our response was likely low because we disseminated the study via email list-servs to a broad cross-section of providers in our selected geographical areas, and we did not offer incentives for participation. We used a convenience sample for this analysis with participants who self-selected to register and complete the survey for an FGM/C training workshop, which could result in a bias related to their interest in the topic of FGM/C. Future studies may expand the geographical area of the study population and utilize a clear sampling frame with prescribed recruiting methods to reduce bias. As discussed previously, the *Empathetic Attitudes* subscale did not have strong reliability as measured by Cronbach’s alpha. A future iteration of the *Empathetic Attitudes* scale should include additional items that broaden the range of attitudes assessed; qualitative research with health care providers would be appropriate to inform further item development. Future item development should consider the behavioral, cognitive, and affective indicators of attitudes that researchers hope to investigate in order to conduct hypothesis testing. Future iterations of the scale should be tested in a random sample of health care providers to avoid selection bias. Our study could have been strengthened by assessing concurrent validity of our subscale using a criterion measure; however, we were not able to identify any validated attitudes or confidence scales to use. Finally, we recognize the limitations inherent in health care provider self-assessment of their confidence for the provision of clinical care, and communication skills, which may be affected by social desirability bias. While confidence, or self-efficacy, is a good indicator of future practice, it is not clear that this confidence translates into improved client experiences. Further research that explores the relationship between health care provider confidence in caring for women affected by FGM/C, and the experiences of the women themselves, could provide insight into the disconnect between provider self-assessment and deficits in quality of care and communication experienced by women living with FGM/C in the diaspora setting.

## Conclusion

To the best of our knowledge, the attitude and confidence scales presented here are the first psychometrically validated measures to assess health care provider attitudes and confidence for the care of women and girls affected by FGM/C. Our confidence scales have strong validity and reliability, our attitudes scales have a clear factor structure, and the *Negative Attitudes* scale has strong reliability. Researchers may explore the relationship between health care provider attitudes and confidence for FGM/C related care with patient outcomes such as patient perceptions of quality of care (including experiences stigma and/or bias), trust in their provider, and receipt of appropriate diagnosis and treatment of FGM/C and associated health complications. Our measures will support future researchers to investigate factors that affect quality of care for women and girls who have experienced FGM/C.

## Supplementary Information

**Additional file 1.** FGC KAP Survey. This file includes the full online survey that was administered to study participants.

## Data Availability

The datasets used and analyzed during the current study are available from the corresponding author on reasonable request.

## Abbreviations

WHO: World Health Organization
FGM/C: Female Genital Mutilation/ Cutting
USA: United States of America
EFA: Exploratory factor analysis
