# Supplementary material for: Assessing the reliability and validity of attitudes and confidence scales for the care of women and girls affected by female genital mutilation/cutting
Source: BMC Public Health. 2021 Jul 17;21:1415. doi: 10.1186/s12889-021-11455-8 (PMC8285805; doi:10.1186/s12889-021-11455-8)
Supplement: Supplementary file 1 — Additional file 1. FGC KAP Survey. This file includes the full online survey that was administered to study participants. [file 12889_2021_11455_MOESM1_ESM.docx]

| **INSTRUCTIONS:**  The answers you provide will help us better understand the knowledge, attitudes, and practices of health care providers, and their educational needs for the care of women and girls affected by female genital cutting. The entire survey should take you approximately 15 minutes. Your answers are confidential and only aggregate or pooled results will be reported.  **ELIGIBILITY:**   - You provide clinical care for women and/or girls - You are 18 years or older - You are a licensed health care provider in current clinical practice | | | | | | | | | | | | | |
| --- | --- | --- | --- | --- | --- | --- | --- | --- | --- | --- | --- | --- | --- |
| **Demographic Information – Please Circle your Response(s)** | | | | | | | | | | | | | |
| **Gender (Circle)**  Female  Male  Transgender  Other | **Race/ Ethnicity**  Black/ African American  White or Caucasian  Latino/ Hispanic  Asian American  Native American/ Indigenous  Other (Specify____________________)  Foreign Born (Specify____________) | | | | | **Clinical Practice**  Resident (PGY_______ Specialty_____________________)  Physician (Specialty_________________________)  Nurse Midwife  Nurse-Practitioner (Specialty______________________)  Physician’s Assistant (Specialty____________________)  Nurse (Specialty___________________)  Social Work  Mental Health (Specify____________________________)  Student  (Specify_____________________________)  Other (Specify______________________________) | | | | | | | |
| **Religious Affiliation**  Muslim  Christian  Hindu  Jewish  Buddhist  Other (Specify______________) | | **Ever Cared for a Woman/ Girl with FGC?**  None  1-3  3-20  >20 | | | **Years in Practice**  <5  5-10  10-20  >20 | | | **Previous FGC Training (select all that apply)**  None  Brief Mention in a Class  Full Class/Lecture During Training  CME/CNE/ CEU Course  Independent learning (read articles)  Trained with FGC-Specialist  Other (Specify___________________) | | | | | |
| **I am affiliated with the following health care facility:______________________________** | | | | | | | | | | | | | |
| **Knowledge and Experience with FGC – Please Circle Your Response(s)** | | | | | | | | | | | | | |
| **Are you aware of the WHO Classification System for FGC?**  YES  NO | | | | **Some circumcised women in the United States avoid health care for pregnancy and/or gyn conditions because they feel (select all that apply):**   1. Stigma from Providers 2. Providers do not know how to care for them due to FGC 3. Legal Concern 4. Fear of Unnecessary Procedures 5. Fear of Death 6. They do not avoid care 7. Unsure | | | | | | | | | |
| **It is illegal to transport a person outside the United States for FGC (vacation cutting)?**  TRUE  FALSE | | | | **FGC performed on a child is considered assault, and therefore reportable as child abuse.**  TRUE  FALSE | | | | | | **I am familiar with laws in my state regarding FGC**  TRUE  FALSE | | | |
| **Defibulation, re-opening the vulvar scar, can be an important intervention to reduce obstetric risk for women with Type 3 FGC (infibulation).**   1. **Have you heard of defibulation before today?**YES     NO 2. **Were you trained on the surgical technique of defibulation?**YES     NO | | | | | | | | | | | |  | |
| **INSTRUCTIONS:**   - **The following table includes potential FGC-related complications that women/girls may experience.** - ***Please Mark X if you were AWARE of the possible complication before today, if not leave blank.*** - ***Please Mark X if you have SEEN the complication in your clinical practice, if not leave blank*** | | | | | | | | | | | |  | |
| **IMMEDIATE POST-FGC COMPLICATIONS** | | | | | | | | | | | |  | |
|  | | | **Aware of Complication?** | | | | **Seen Complication in My Practice?** | | | | | **If yes you have seen, in which country?** | |
| Hemorrhage | | |  | | | |  | | | | |  | |
| Pain | | |  | | | |  | | | | |  | |
| Hemorrhagic, neurogenic or septic shock | | |  | | | |  | | | | |  | |
| Genital tissue swelling | | |  | | | |  | | | | |  | |
| Genital and reproductive tract infections | | |  | | | |  | | | | |  | |
| Urinary tract infections | | |  | | | |  | | | | |  | |
| Acute urinary retention | | |  | | | |  | | | | |  | |
| Dysuria | | |  | | | |  | | | | |  | |
| Damage to urethra and adjacent tissues | | |  | | | |  | | | | |  | |
| Death | | |  | | | |  | | | | |  | |
| **FGC-RELATED OBSTETRIC COMPLICATIONS** | | | | | | | | | | | |  | |
|  | | | **Aware of Complication?** | | | | **Seen Complication in My Practice?** | | | | | **If yes you have seen, in which country?** | |
| Cesarean section | | |  | | | |  | | | | |  | |
| Postpartum hemorrhage | | |  | | | |  | | | | |  | |
| Episiotomy | | |  | | | |  | | | | |  | |
| Prolonged labor | | |  | | | |  | | | | |  | |
| Obstetrical tears/lacerations | | |  | | | |  | | | | |  | |
| Instrumental delivery | | |  | | | |  | | | | |  | |
| Labor dystocia | | |  | | | |  | | | | |  | |
| Extended maternal hospital stay | | |  | | | |  | | | | |  | |
| Stillbirth or early neonatal death | | |  | | | |  | | | | |  | |
| Infant resuscitation at delivery | | |  | | | |  | | | | |  | |
| **FGC- RELATED SEXUAL COMPLICATIONS** | | | | | | | | | | | |  | |
|  | | | **Aware of Complication?** | | | | **Seen Complication in My Practice?** | | | | | **If yes you have seen, in which country?** | |
| Decreased lubrication during sexual intercourse | | |  | | | |  | | | | |  | |
| Reduced frequency of orgasm or anorgasmia | | |  | | | |  | | | | |  | |
| Dyspareunia (pain with sex) | | |  | | | |  | | | | |  | |
| Decreased sexual satisfaction | | |  | | | |  | | | | |  | |
| Reduced sexual desire and arousal | | |  | | | |  | | | | |  | |
| **FGC- RELATED PSYCHOLOGICAL COMPLICATIONS** | | | | | | | | | | | |  | |
|  | | | **Aware of Complication?** | | | | **Seen Complication in My Practice?** | | | | | **If yes you have seen, in which country?** | |
| PTSD | | |  | | | |  | | | | |  | |
| Anxiety | | |  | | | |  | | | | |  | |
| Depression | | |  | | | |  | | | | |  | |
| **FGC-RELATED LONG TERM COMPLICATIONS** | | | | | | | | | | | |  | |
|  | | | **Aware of Complication?** | | | | **Seen Complication in My Practice?** | | | | | **If yes you have seen, in which country?** | |
| Chronic vulvar and clitoral pain | | |  | | | |  | | | | |  | |
| Recurrent genital tract infections | | |  | | | |  | | | | |  | |
| Dysmenorrhea | | |  | | | |  | | | | |  | |
| Recurrent UTIs | | |  | | | |  | | | | |  | |
| Urinary obstruction | | |  | | | |  | | | | |  | |
| **INSTRUCTIONS:**   - **The following statements reflect different attitudes toward the practice of Female Genital Cutting, and the people who practice Female Genital Cutting.** - **Read each statement carefully, and MARK X in the box to indicate whether you *Strongly Agree, Agree, Disagree, or Strongly Disagree.*** | | | | | | | | | | | | | |
| **ATTITUDES TOWARDS THE PRACTICE OF FGC** | | | | | | | | | | | | | |
|  | | | | | | | | **Strongly Agree** | **Agree** | | **Disagree** | | **Strongly Disagree** |
| FGC is a violation of human rights | | | | | | | |  |  | |  | |  |
| Symbolic nicking or cutting of the female genitalia is an effective way to reduce the harm of FGC compared to more extensive procedures | | | | | | | |  |  | |  | |  |
| Health Care Providers who perform any form of FGC, including symbolic nicking, should be charged with a crime | | | | | | | |  |  | |  | |  |
| Adult women have the right to undergo FGC | | | | | | | |  |  | |  | |  |
| Parents have the right to have their daughters circumcised (undergo FGC) | | | | | | | |  |  | |  | |  |
| Cultural humility (the ability to maintain an interpersonal stance that is other-oriented (or open to the other) in relation to aspects of cultural identity that are most important to the [patient])  of the health care provider is an important factor in reducing adverse outcomes for FGC-affected women and girls | | | | | | | |  |  | |  | |  |
| Health care providers should perform reinfibulation (re-closing of the vulvar scar following childbirth) if the woman requests it | | | | | | | |  |  | |  | |  |
| **ATTITUDES TOWARD THOSE WHO PRACTICE FGC** | | | | | | | | | | | | | |
|  | | | | | | | | **Strongly Agree** | **Agree** | | **Disagree** | | **Strongly Disagree** |
| Communities that practice FGC are oppressive towards women | | | | | | | |  |  | |  | |  |
| Communities that practice FGC are honoring an important cultural tradition | | | | | | | |  |  | |  | |  |
| Parents who have their daughter circumcised are abusing them | | | | | | | |  |  | |  | |  |
| Parents who have their daughter circumcised are protecting her future marriage prospects | | | | | | | |  |  | |  | |  |
| Women who have undergone FGC are empowered agents | | | | | | | |  |  | |  | |  |
| Women who have undergone FGC are victims of an oppressive cultural practice | | | | | | | |  |  | |  | |  |
| **INSTRUCTIONS:**   - **The following statements ask you to reflect on how CONFIDENT you feel in regards to different skills needed to care for women and girls affected by FGC.** - **Read each statement carefully, and MARK X in the box to indicate whether you *Strongly Agree, Agree, Disagree, or Strongly Disagree*with the statement*.*** | | | | | | | | | | | | | |
| **Health Care Providence Confidence Caring for Women and Girls Affected by FGC** | | | | | | | | | | | | | |
|  | | | | | | | | **Strongly Agree** | **Agree** | | **Disagree** | | **Strongly Disagree** |
| On inspection of the female genitalia, I can identify a woman with FGC | | | | | | | |  |  | |  | |  |
| On identification of a woman with FGC, I can assign the appropriate WHO Type classification | | | | | | | |  |  | |  | |  |
| On identification of a woman with FGC, I can appropriately code a visit to document the presence and type of FGC using ICD-10 and CPT codes | | | | | | | |  |  | |  | |  |
| Conduct an effective reproductive/sexual health history via an interpreter | | | | | | | |  |  | |  | |  |
| Respond to the health concerns of women with FGC by engaging in non-judgmental listening | | | | | | | |  |  | |  | |  |
| Counsel women on the possible complications she may experience related to FGC | | | | | | | |  |  | |  | |  |
| Discuss defibulation with pregnant women who have undergone Type 3 FGC in a culturally sensitive manner | | | | | | | |  |  | |  | |  |
| Perform defibulation of an FGC-related vulvar scar | | | | | | | |  |  | |  | |  |
| Perform defibulation during the second stage of labor | | | | | | | |  |  | |  | |  |
| Respond to a request for reinfibulation (re-closing of the vulvar scar following childbirth) with cultural humility | | | | | | | |  |  | |  | |  |
| Create a positive therapeutic relationship with a patient who is refuses a recommended procedure | | | | | | | |  |  | |  | |  |
